# AGO6 Functions in RNA-Mediated Transcriptional Gene Silencing in Shoot and Root Meristems in *Arabidopsis thaliana*

**DOI:** 10.1371/journal.pone.0025730

**Published:** 2011-10-05

**Authors:** Changho Eun, Zdravko J. Lorkovic, Ulf Naumann, Quan Long, Ericka R. Havecker, Stacey A. Simon, Blake C. Meyers, Antonius J. M. Matzke, Marjori Matzke

**Affiliations:** 1 Gregor Mendel Institute, Austrian Academy of Sciences, Vienna, Austria; 2 Department of Plant Sciences, University of Cambridge, Cambridge, United Kingdom; 3 Department of Plant and Soil Sciences, and Delaware Biotechnology Institute, University of Delaware, Newark, United States of America; Oregon State University, United States of America

## Abstract

RNA-directed DNA methylation (RdDM) is a small interfering RNA (siRNA)-mediated epigenetic modification that contributes to transposon silencing in plants. RdDM requires a complex transcriptional machinery that includes specialized RNA polymerases, named Pol IV and Pol V, as well as chromatin remodelling proteins, transcription factors, RNA binding proteins, and other plant-specific proteins whose functions are not yet clarified. In *Arabidopsis thaliana*, DICER-LIKE3 and members of the ARGONAUTE4 group of ARGONAUTE (AGO) proteins are involved, respectively, in generating and using 24-nt siRNAs that trigger methylation and transcriptional gene silencing of homologous promoter sequences. AGO4 is the main AGO protein implicated in the RdDM pathway. Here we report the identification of the related AGO6 in a forward genetic screen for mutants defective in RdDM and transcriptional gene silencing in shoot and root apical meristems in *Arabidopsis thaliana.* The identification of AGO6, and not AGO4, in our screen is consistent with the primary expression of AGO6 in shoot and root growing points.

## Introduction

RNA-directed DNA methylation is a small interfering RNA (siRNA)-mediated epigenetic modification that is highly developed in flowering plants. RdDM is characterized by *de novo* methylation of cytosines in all sequence contexts (CG, CHG and CHH, where H is A, T or C) within the region of siRNA-DNA sequence homology. Forward genetic screens in *Arabidopsis thaliana* have revealed that RdDM requires a specialized transcriptional machinery comprising two RNA polymerase II (Pol II)-related RNA polymerases called Pol IV and Pol V, as well as SNF2 chromatin remodelling proteins, various putative transcription factors, RNA binding proteins, histone modifying enzymes, and several novel, plant-specific proteins whose functions are not yet well understood [1]–[3]. Pol IV is involved in producing the siRNA trigger for RdDM whereas Pol V acts downstream of siRNA production to facilitate *de novo* methylation of homologous DNA sequences. To accomplish this, Pol V is thought to synthesize scaffold transcripts that interact with the complementary siRNA trigger, leading to recruitment of the methylation machinery at the target DNA site [4]. Two independent forward genetic screens have recently confirmed that DRM2 is the major DNA cytosine methyltransferase acting in the RdDM pathway [5], [6].

Transposons and repeated sequences are frequent targets of RdDM [7], [8]. However, mutants defective in RdDM do not ordinarily unleash transposon activity. By contrast, the methylation-defective mutant *ddm1* transcriptionally derepresses and mobilizes many transposons [9], [10]. Nevertheless, recent studies suggest that components of the RdDM pathway operate on specific retrotransposon families to block transcriptional activation and mobilization in the DNA methyltransferase mutant *met1* [11] and to prevent stress-induced transposition across generations [12].

Core proteins of the RNA interference machinery, DICER-LIKE (DCL) and ARGONAUTE (AGO), play key roles in the RdDM pathway by generating and using siRNAs that guide methylation of homologous DNA sequences. Forward genetic screens have verified that DCL3, one of four DCL activities in *Arabidopsis*, is responsible for processing double-stranded RNA precursors into 24-nt siRNAs that induce RdDM [5], [13]. AGO proteins function in RNA silencing effector complexes by binding the 3′ and 5′ ends of the guide siRNAs at their N-terminal PAZ domain and MID domain, respectively. In addition, many AGO proteins cleave target RNAs at the siRNA complementary site by means of an endonuclease (‘slicer’) activity in their C-terminal PIWI domain, which structurally resembles RNaseH [14]–[16]. AGO4 is the major AGO protein implicated in RdDM, as indicated by the recovery of *ago4* mutations in several independent forward genetic screens [5], [17], [18]. By contrast, the related AGO6 has been assigned a less prominent role in RdDM, having been retrieved so far in only one forward genetic screen [19]. Here we report the identification of AGO6 in a forward genetic screen for mutants defective in RdDM and transcriptional gene silencing in shoot and root apical meristems. We propose that the identification of exclusively AGO6, and not AGO4, in our screen reflects the primary expression of AGO6 in shoot and root apices.

## Results

**Figure 1:**
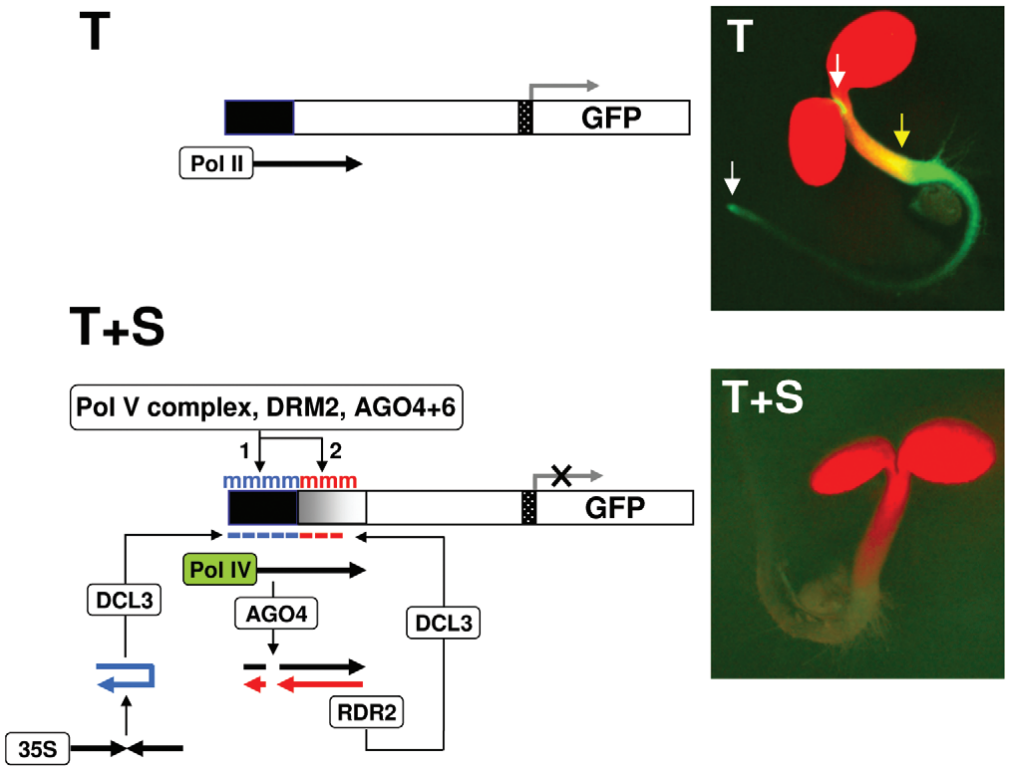
Transgene silencing system and step-wise establishment and spread of RdDM. The two-component transgene silencing system consists of a target locus (T) containing a minimal promoter (dotted box) fused to an upstream enhancer (black box), which drives the expression of a *GFP* reporter gene in shoot and root apical meristem regions (SAM and RAM, respectively) (white arrows, image T). *GFP* expression in the hypocotyl region is also seen at this stage of development (yellow arrow, image T). The silencer locus (S) contains an inverted repeat of upstream enhancer sequences under the control of the cauliflower mosaic virus 35S promoter. The resulting hairpin RNA (blue) is processed by DCL3 into 24-nt primary siRNAs (blue dashes), which induce methylation of the target enhancer and silencing of the *GFP* gene (image T+S). In a current hypothetical model, the presence of primary methylation provokes a polymerase switch such that Pol IV replaces Pol II in transcribing a non-coding ‘nascent’ RNA (black arrow) that initiates in the target enhancer region (T+S) [13]. The nascent Pol IV transcript is cleaved by an AGO protein (most likely AGO4; this work), producing RNA fragments that provide substrates for RDR2. The resulting dsRNA is processed by DCL3 into 24-nt secondary siRNAs (red dashes), which foster spreading of methylation into the downstream region (shaded gray box; red m). Primary (blue) and secondary (red) RdDM both require the action of the Pol V complex and the *de novo* methyltransferase DRM2, as well as either AGO6 in RAM and SAM regions (this study) or the redundant action of AGO4 and AGO6 in leaves [13].

We established a two-component transgene system designed to identify factors important for RdDM and transcriptional gene silencing in a developmental context in *Arabidopsis thaliana* ecotype Col-0 [13], [20]. The system consists of a target locus, which contains a *GFP* reporter gene under the control of an upstream enhancer that is active in shoot and root meristems, and an unlinked silencer locus, which encodes a hairpin RNA homologous to target enhancer sequences. Processing of the hairpin RNA by DCL3 produces 24-nt siRNAs, which trigger methylation of the target enhancer, resulting in transcriptional silencing of the *GFP* gene (Fig. 1).

Screens to identify mutants defective in RdDM and *GFP* silencing were carried out as described previously [13], [20]. Briefly, following mutagenesis of M1 seeds by ethyl methanesulfonate (EMS), mutants were identified by screening the root tips of M2 seedlings (the first generation when an EMS-induced recessive mutation can be homozygous) for reactivation of *GFP* expression in the presence of the silencer locus. So far, eight *dms* (defective in meristem silencing) mutants (*dms1* to *dms8*) have been identified in this screen (Table 1).

**Table 1:** Defective in meristem silencing (*dms*) mutants.

Mutant Name	Common Name and AGI number	Number of alleles	Description
DMS1	DRD1 (At2g16390)	23	SNF2-like chromatin remodeling protein (Kanno *et al.* 2008)
DMS2	NRPD2a (At3g23780)	24	common second largest subunit of Pol IV and Pol V (Kanno *et al.* 2008)
DMS3	DMS3 (At3g49250)	10	structural maintenance of chromosomes hinge domain-containing protein (Kanno *et al.* 2008)
DMS4	DMS4 (At2g30280)	2	IWR1 putative transcription factor (Kanno *et al.* 2010)
DMS5	NRPE1 (At2g40030)	26	unique largest subunit of Pol V (Kanno *et al.* 2008)
DMS6	DCL3 (At3g43920)	2	Dicer-like3 (Daxinger *et al.* 2009)
DMS7	RDM1 (At3g22680)	5	plant specific small protein with novel fold (Gao *et al.* 2010)
DMS8	DRM2 (At5g14620)	3	*de novo* DNA methyltransferase (Naumann *et al.* 2011)
DMS9	AGO6 (At2g32940)	4	Argonaute6 (this study)

From the forward genetic screen for mutants defective in RdDM and TGS of a *GFP* reporter gene in root and shoot meristems, we originally retrieved 115 mutants that were fertile and could be propagated. The mutated gene has been identified in 99 mutants that can be placed into nine complementation groups - DMS1 to DMS9 - which are described here by their common name, Arabidopsis Genome Initiative (AGI) number and function in the RdDM pathway. The number of alleles obtained for each mutant is shown in third column. Four mutants contain structural rearrangements of the silencer locus, leading to loss of *GFP* silencing in the absence of a second site mutation (not shown). The remaining, unidentified mutants do not correspond to DMS1 to DMS9 or to AGO4.

**Figure 2:**
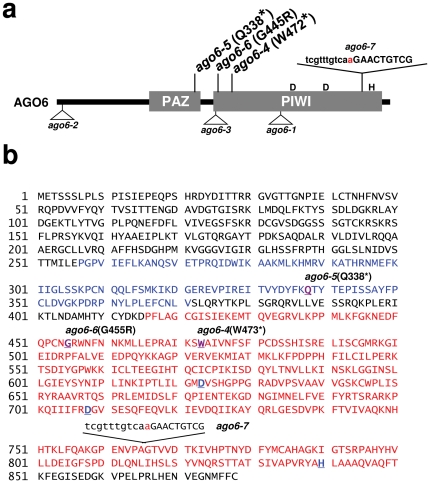
AGO6 amino acid sequence and positions of mutations. **a**) Schematic presentation of AGO6 domains and mutations. The EMS-induced point mutations identified in this study (*ago6-4* to *ago6-7*) are indicated at the top of the figure. The DDH triad comprises the catalytic center of the PIWI slicer domain. Three T-DNA insertion mutants (bottom) have been described previously: *ago6-1* [19]; *ago6-2* (Salk_031553) [19]; *ago6-3* (Salk_106607) [22]. **b**) The AGO6 protein is 879 amino acids in length. The PAZ and PIWI domains are highlighted in blue and red letters, respectively. The positions of the mutations in *ago6-4*, *ago6-5*, *ago6-6* and *ago6-7* alleles are indicated. The DDH residues comprising the catalytic core of the RNA slicer activity in the PIWI domain are also shown in blue underlined letters. The G to A mutation in the 3′ splice site of the *ago6-7* allele is highlighted.

To determine the mutated gene in the next mutant in the series, *dms9*, we carried out whole genome sequencing on DNA isolated from *dms9* mutant plants and examined the consensus sequence for mutations in genes encoding known chromatin factors (http://www.chromdb.org/). This analysis revealed a mutation in the gene encoding AGO6 (At2g32940), one of ten AGO proteins in *Arabidopsis* [15], [21]. Sequencing the *AGO6* gene in several other uncharacterized *dms* mutants revealed three additional *ago6* alleles. In view of previously studied *ago6* mutations - *ago6-1* to *ago6-3*, which contain T-DNA insertions [19], [22] - we designated the four new alleles *ago6-4* to *ago6-7* (Fig. 2a). The *ago6-4* and *ago6-5* alleles contain premature stop codons; *ago6-6* has an amino acid substitution in a conserved glycine residue in the PIWI domain; and *ago6-7* contains a 3′ splice site mutation (G to A) toward the end of the gene, resulting in a truncated protein that lacks the terminal histidine (H) residue of the arginine (D)-arginine-histidine (DDH) catalytic triad in the PIWI domain (Fig. 2a and b).

We carried out a complementation analysis by transforming the *ago6-4* mutant with either a wild type *AGO6* cDNA or a mutated version in which the first amino acid of the DDH catalytic triad is changed to alanine (D623A) (Fig. 2a and b). At least 20 progeny from transgenic lines homozygous for a single copy of the respective construct (three independent lines for each construct) were examined under a fluorescence microscope for *GFP* expression in the SAM and RAM approximately 3 weeks after germination. Only the wild type cDNA complemented the *ago6-4* mutation and restored *GFP* silencing (data not shown).

**Figure 3:**
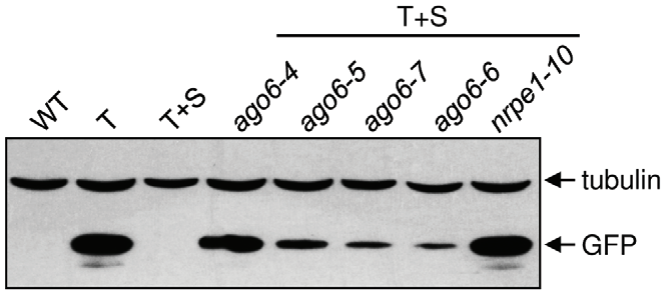
Western blot analysis of GFP protein in *ago6* mutants. Accumulation of GFP protein was observed in all four *ago6* mutants as well as *nrpe1-10* (defective in the largest subunit of Pol V), demonstrating release of *GFP* silencing. Control lanes include non-transgenic wild type plants (WT) and transgenic plants containing only the target locus (T) or the target and silencer loci (T+S). All mutants are in a T+S background. Levels of a constitutively expressed tubulin protein are shown as a loading control.

**Figure 4:**
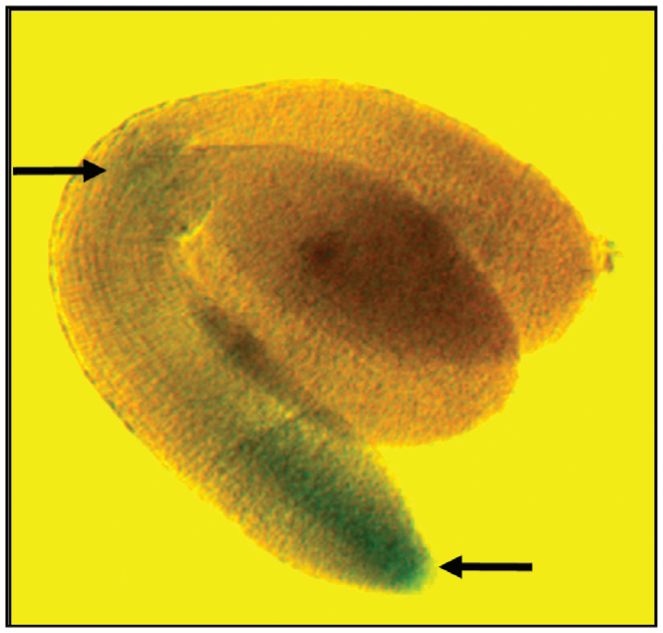
*AGO6* promoter is active in the shoot and root meristem regions. In the mature embryo shown here, a *GUS* reporter gene under the control of the AGO6 promoter shows weak but detectable expression in the shoot and root apical meristems (arrows) and the connecting vasculature.

The *ago6* mutants we identified are generally fertile and do not show obvious developmental abnormalities. However, the extent to which *GFP* silencing was alleviated varied for each allele. The two alleles containing premature stop codons within and just beyond the PAZ domain (*ago6-5* and *ago6-4,* respectively) conditioned the strongest release of *GFP* silencing, as assessed by GFP protein levels on Western blots, although the reactivation was still somewhat less than in a mutant defective in the largest subunit of Pol V (*nrpe1*) (Fig. 3). GFP silencing was released to a lesser extent with the other two alleles, *ago6-7* and *ago6-6* (Fig. 3). Consistent with the GFP protein data, GFP fluorescence could be observed under a fluorescence microscope in both the shoot and root meristems in seedlings containing the strong *ago6-4* allele, whereas GFP fluorescence was more variable and primarily visible in the hypocotyl region (Fig. 1) of seedlings containing the weaker alleles (data not shown). As reported previously [23], the expression of a *GUS* reporter gene under the control of the *AGO6* promoter in mature embryos was generally concentrated in the shoot and root apices and the vasculature in between these two regions (Fig. 4).

**Figure 5:**
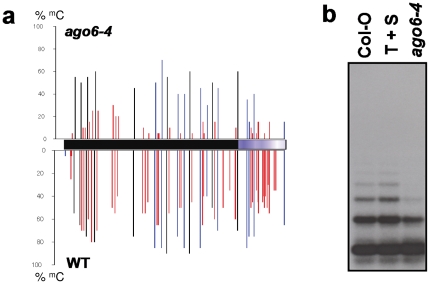
Methylation analysis. **a**) Bisulfite sequence analysis of methylation at the target enhancer (black bar) and downstream region (blue bar) in rosette leaves of wild type T+S plants and the *ago6-4* mutant. Approximately 20 clones were sequenced for each genotype. The percent methylation at each cytosine residue is indicated by the vertical lines: CG (black), CHG (blue); CHH (red). **b**) Southern blot analysis of methylation in 5S rDNA repeats as assessed by digestion with *Hae*III, which reports on CHH methylation. Non-transgenic wild type plants (Col-0) and wild type T+S plants are shown as controls. More complete digestion in the *ago6-4* mutants indicates loss of CHH methylation.

We molecularly characterized the *ago6-4* mutation by examining DNA methylation of target sequences and siRNA accumulation. DNA methylation was assayed in rosette leaves and siRNA accumulation determined in mixed-stage floral inflorescences. At the target transgene enhancer, methylation of cytosines in all sequence contexts was reduced 2.8-fold in the *ago6-4* mutant (Fig. 5a), which is consistent with the release of *GFP* silencing in meristem regions. Endogenous 5S rDNA repeats also displayed decreased CHH methylation in the *ago6-4* mutant (Fig. 5b).

**Figure 6:**
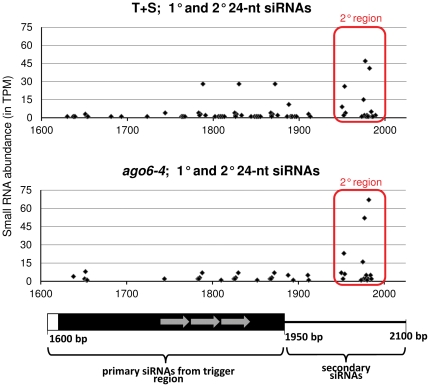
Analysis of 24-nt siRNAs from wild type T+S and *ago6-4* inflorescences. Abundance of primary and secondary 24-nt siRNAs derived from the target enhancer and downstream region, respectively, as measured in two SBS libraries, one each from wild type T+S plants and the *ago6-4* mutant. The Y axis indicates the normalized abundance of each matching siRNA in Transcripts per Million (TPM). Positions of these small RNAs within the primary and secondary siRNA regions are indicated by the X axis, relative to the region of 1600 to 2100 bp in the target transgene construct (Accession number HE582394). A short tandem repeat comprising three copies of a ∼ 41 bp monomer in the primary trigger region is demarcated with grey arrows [20]. The region of the target transgene generating secondary siRNAs is indicated by a red box.

Several populations of siRNA are produced in silenced plants. Hairpin-derived primary siRNAs are 21-, 22-, and 24-nt in length due to the redundant action of multiple DCL enzymes, but only the 24-nt size class produced by DCL3 is required for target enhancer methylation and *GFP* silencing in our system [13]. Secondary siRNAs, which are 24-nt in length and dependent on Pol IV and RNA-DEPENDENT RNA POLYMERASE2 (RDR2) for their biogenesis, promote spreading of methylation downstream of the target enhancer region (Fig. 1, T+S), but they are not required for *GFP* silencing [13]. As shown by deep sequencing of small RNAs, primary and secondary 24-nt siRNAs accumulate in the *ago6-4* mutant (Fig. 6). Of particular interest is the observation of nearly wild type levels of secondary siRNAs in *ago6-4*, since these were largely eliminated in the *ago4-1* mutant [13] and in other Pol V pathway mutants such as *drd1*, *nrpe1*, *nrpd2a*, *dms3* and *dms4* [20], [24]. The presence of at least some CHH methylation in the downstream region is consistent with the presence of secondary siRNAs (Fig 5a).

## Discussion

We have identified AGO6 in a screen for mutants defective in RdDM and transcriptional gene silencing in root and shoot meristem regions in *Arabidopsis thaliana* ecotype Col-0. The four *ago6* alleles we describe are the first loss-of-function point mutations identified in the *AGO6* gene. AGO6 belongs to the AGO4 group of AGO proteins that is specialized for siRNA-mediated chromatin modifications [3], [15]. This group includes the founding member *AGO4*, which is widely expressed and most frequently implicated in RdDM [17], [23]; *AGO6*, which shows restricted expression in root and shoot growing points and in the vasculature connecting these regions [23]; *AGO9*, which is expressed in ovules, anthers and seed coat [25], [26]; and *AGO8*, which is a pseudogene of *AGO9* [21].

Both *AGO6* [19] and *AGO4* [5], [17], [18] have been identified in previous forward genetic screens and they are generally thought to act redundantly in the RdDM pathway. AGO4, however, is usually afforded the more prominent role in RdDM. For example, in the transgene silencing system used in the forward screen that first identified *AGO6*, an introgressed *ago4-1* mutation was found to have a stronger suppressing effect on the phenotype than mutations in *AGO6* [19]. Accordingly, an *ago4* mutation was later identified in the same screen [18]. Although the *ago4-1* mutation releases silencing partially and non-uniformly when introgressed into our silencing system [13], *ago4* mutations are not likely to be recovered in our forward screen. Multiple alleles have been identified for all nine *dms* mutants characterized so far (Table 1), indicating the screen is close to saturation, yet sequence analysis has demonstrated that none of the remaining unidentified *dms* mutants has a point mutation in the *AGO4* gene (Z. Lorkovic, U. Naumann, M. Matzke; unpublished results). These findings suggest that AGO6, but not AGO4, plays a critical role in RdDM that leads to transcriptional gene silencing in our system.

The exclusive identification of AGO6 in our screen may reflect the distinct expression patterns of AGO proteins. As mentioned above, *AGO4* is ubiquitously expressed, whereas the expression of *AGO6* is largely confined to the shoot and root growing points and the connecting vascular tissue, shown here and previously [23]. In view of this expression pattern, it is understandable that we retrieved *AGO6* because mutants were identified by screening for reactivation of *GFP* expression in root tips of seedlings. The only other forward genetic screen to identify mutations in AGO6 assessed the recovery of luciferase activity in 12-day-old seedlings [19], which may have enhanced the ability to visualize reactivation in meristems.

Another factor that may explain our findings concerns the molecular requirements of our silencing system. Because the 24-nt primary siRNAs are produced from a hairpin RNA transcribed by Pol II, RdDM of the target enhancer and *GFP* silencing are independent of Pol IV pathway components such as NRPD1, the largest subunit of Pol IV, and RDR2 [13], which are normally needed to produce double stranded RNA precursors of siRNAs [27]. Therefore, silencing and methylation in our system depend only on Pol V pathway members, which are directly involved in the process of *de novo* DNA methylation downstream of siRNA production [1]–[3]. It follows that the components identified in our forward genetic screen, including AGO6, act in the Pol V-mediated steps of RdDM. By contrast, other forward genetic screens that have retrieved *ago4* mutants have identified components of both the Pol IV and Pol V pathways, suggesting the involvement of AGO4 in both Pol IV and Pol V functions [5], [18]. One interpretation of these results is that AGO6 is the major AGO protein associated with Pol V-mediated *de novo* methylation in root and shoot meristems, whereas in other cell types, AGO6 acts redundantly in *de novo* methylation with AGO4, which contributes in addition to Pol IV-dependent functions (discussed further below). Consistent with this idea, methylation of the target transgene enhancer in leaves was reduced not only in the *ago6-4* mutant (this study) but also after introgressing the *ago4-1* mutation into the target/silencer line [13].

Another point to consider is the role of AGO6 in the RdDM mechanism. Owing to the presence of several domains, AGO proteins have multiple functions in siRNA-mediated silencing pathways. AGO proteins bind the guide siRNA at their PAZ and MID domains (‘guide’ function), and some have the ability to cleave target RNAs through an endonuclease (‘slicer’) activity in their PIWI domain. Whereas the guide function of AGO proteins in RdDM seems straightforward, the contribution of the RNA slicer activity to RdDM is less obvious. In this context, the *ago6-7* allele is informative because it encodes a truncated protein that lacks the terminal histidine residue of the DDH catalytic triad that is necessary (but not sufficient) for the RNA slicer activity [14], [15]. Although it is not yet known whether AGO6 is cleavage-competent, the recovery of the *ago6-7* allele suggests that the RNA slicer activity of AGO6 is important for RNA-mediated transcriptional gene silencing in shoot and root meristems, at least at the DNA targets tested in this study. This idea is further supported by our complementation experiments, in which a wild type *AGO6* cDNA but not one with a mutation in the DDH catalytic triad complemented the *ago6* mutant phenotype by restoring *GFP* silencing in meristem regions.

Why is the RNA slicer activity of AGO6 necessary for RdDM? The slicer activity of AGO proteins is clearly important in cases of post-transcriptional gene silencing that involve siRNA-guided cleavage of target mRNAs [15]. In the RdDM pathway, slicer activity is probably needed for the production of secondary siRNAs, which most likely requires cleavage of an aberrant Pol II or Pol IV transcript to generate fragments that are substrates for RDR2 (Fig. 1) [13], [28]. In our silencing system, the putative slicer activity of AGO6 is probably not involved extensively in secondary siRNA formation because secondary siRNAs still accumulate in the *ago6-4* mutant. By contrast, secondary siRNAs were undetectable on Northern blots after introgressing the *ago4-1* mutation into the target/silencer line [13]. Thus it is likely that AGO4 provides the primary slicer activity for Pol IV-dependent secondary siRNA formation in our silencing system (Fig. 1). As shown previously in *Caenorhabditis elegans*, a further function of the RNA slicer activity of AGO proteins is the removal of the passenger strand from the siRNA duplex that is originally incorporated into AGO proteins, leaving behind only the guide siRNA strand in the mature silencing effector complex [16], [29]. Similarly, the putative slicer activity of AGO6 is likely needed to remove the passenger strand from the small RNA duplex, permitting the remaining guide strand to function efficiently in RdDM.

In summary, our identification of exclusively AGO6 in our forward genetic screen suggests that it is the main AGO4 group protein acting in the Pol V-mediated steps of RNA-mediated transcriptional gene silencing in shoot and root meristems. Despite the importance of AGO6 in these meristem regions, the *ago6* mutants we identified do not show obvious developmental phenotypes over the generations analyzed in this study. Additional work is required to assess the consequences of AGO6 deficiency for transposon silencing, genome integrity, and gene expression in meristem regions.

## Materials and Methods

### Plant material, complementation analysis, and visualization of GFP

The *ago6* mutants described in this paper are in the Col-0 background. Plants were grown in a growth chamber set at 21°C and a light regime of 16 hours light/8 hours dark. For complementation analyses, wild type and ‘DDH’ mutant versions of the *AGO6* cDNA under the control of the native *AGO6* promoter were introduced into the *ago6-4* mutant using the floral dip method [30]. Details of the constructs are available from the authors on request. For each construct, we obtained three independent lines containing single copies of the respective construct. After breeding to homozygosity, progeny were assessed microscopically for *GFP* expression approximately three weeks post-germination. GFP fluorescence was visualized in seedlings using a Leica stereofluorescence microscope MZFLIII. Seedlings were grown under sterile conditions on solid Murashige and Skoog (MS) medium in a 23°C incubator with a 16 h light/8 h dark cycle.

### CTAB procedure to isolate root DNA for whole genome sequencing

Approximately two-week old mutant seedlings germinated on solid MS medium were added to 150 ml sterile liquid MS medium in a 500 ml Erlenmeyer flask, and the flask shaken at low speed (100 rpm) on a rotary shaker at room temperature. After several weeks of root growth, approximately 1.5 g of roots were harvested free of green material, frozen in liquid nitrogen, and stored at −80°C until use. Nuclear DNA was isolated from roots using a modified CTAB (cetyltrimethylammonium bromide) procedure [31]. Frozen roots were ground in liquid nitrogen using a mortar and pestle. The resulting slurry was poured into a 50 ml Nalgene tube and after the liquid nitrogen evaporated 15 ml of CTAB buffer (140 mM sorbitol, 220 mM Tris-HCl, pH 8.0, 22 mM EDTA, 800 mM NaCl, 1% sodium sarkosyl, 0.8% CTAB) were added. The tube was capped and vortexed (with regular venting) until a smooth suspension formed. The tube was incubated in a 65°C water bath for 20 minutes with occasional vortexing. Then one volume (15 ml) of chloroform was added to the suspension and the tube was placed on a mixing wheel for 20 minutes followed by centrifugation at 3800 rpm (Sorvall HB4 rotor) for 5 minutes to resolve the phases. The top phase was transferred to a fresh Nalgene tube and one volume of cold isopropanol was added. After gentle mixing, the tube was placed on ice for 30 minutes then centrifuged as above. The liquid was decanted completely and the pellet dissolved in 3 ml of 0.1 X TE (1 mM Tris/HCl pH 8 and 0.1 mM EDTA). To remove RNA, 15 µl of DNase-free RNase (Roche Diagnostics GmbH, Vienna, Austria) was added and incubated at 37°C for 20 minutes. Then, 3 ml 4M lithium acetate were added and the tube was incubated on ice for 20 minutes, followed by centrifugation as above. The supernatant was poured into a fresh Nalgene tube, 12 ml 100% ethanol were added and the tube placed on ice for 20 minutes followed by centrifugation as above. After pouring off the supernatant, the pellet was dissolved overnight in 1 ml sterile distilled water at 4°C.

The next day, the DNA solution was phenol extracted and the DNA ethanol precipitated. The DNA pellet was washed with 70% ethanol. After centrifugation, residual ethanol was removed and the pellet dried under vacuum for 5 minutes. The pellet was dissolved overnight in 120 µl sterile distilled water at 4°C. The DNA concentration should be around 1 µg/µl. DNA was sonicated using a Covaris S2 (Covaris, Inc., Woburn, Massachusetts, USA) to produce fragments approximately 300–800 bp in length for making sequence libraries for paired end reads.

### Whole genome sequencing

Paired end sequencing of a genomic DNA library was performed with 72 bp read length using an Illumina Genome Analyzer II (GAII); these genomic re-sequencing data are available upon request. For the raw data analysis we used BWA 0.5.6. [32] for gapped alignment (with maximal 4 mismatches and 1 gap) and SAMtools 0.1.6 [33] for single nucleotide polymorphism (SNP) calls.

### Protein extraction, SDS-PAGE and Western blotting

Approximately 50 mg of three week old seedlings grown on MS medium were frozen in liquid nitrogen, disrupted using a mortar and pestle and resuspended in 100 µl of extraction buffer (50 mM HEPES-KOH pH 7.9, 400 mM KCl, 2.5 mM MgCl_2_, 1 mM EDTA, 1 mM DTT, 0.1% Triton X-100) supplemented with EDTA-free protease inhibitor cocktail (Roche Diagnostics GmbH, Vienna, Austria). The suspension was vortexed three times for 15 sec and centrifuged for 10 min in an Eppendorf centrifuge at maximum speed at 4°C. The supernatants were mixed with equal volumes of extraction buffer without KCl. Proteins were separated by SDS-PAGE (10% gel), transferred to a PVDF membrane (Millipore, Vienna Austria), followed by Western blotting according to standard procedures. Mouse anti-tubulin (Sigma-Aldrich, Vienna, Austria), and mouse anti-GFP (Roche Diagnostics GmbH, Vienna, Austria) monoclonal antibodies were used at 1∶1,000 dilutions. Secondary antibody, goat anti-mouse IgG-conjugated with horseradish peroxidase (Biorad, Vienna, Austria), was used at 1∶10,000 dilution. The blots were developed using an enhanced chemoluminescence kit (AmershamPharmacia Biotech, Freiburg, Germany).

### Small RNA sequencing

Libraries of small RNAs were constructed from total RNA isolated from mixed stage inflorescence tissues of the transgenic wild type control and the *ago6-4* mutant using TRIzol reagent (Invitrogen). Total RNA (200 ug) for each sample was used to construct the small RNA libraries as described previously [34] but with different adapters. The RNA oligos (Dharmacon) used for small RNA ligations were as follows: 5′ RNA Adapter (5′OH-GUUCAGAGUUCUACAGUCCGACGAUC-OH 3′) and 3′ RNA Adapter: (5′ pUCGUAUGCCGUCUUCUGCUUGUidt 3′; p, phosphate; idT, inverted deoxythymidine). Libraries were sequenced on an Illumina GAII at the Delaware Biotechnology Institute. This generated 4,510,985 total reads from wild type (with trigger and silencer transgenes) and 2,327,100 from the *ago6-4* mutant. The small RNA sequence data are available from NCBI's Gene Expression Omnibus (GEO) and are accessible via GEO Series accession number GSE28537.

Adapter sequences were removed using a Perl script, generating small RNA sequences plus abundances. The data were matched to the Arabidopsis genome (TAIR v9) as well as the transgenes described previously [20]. Accession numbers of the target and silencer sequences are HE582394 and HE584556, respectively. Of the 4,510,985 total reads from transgenic wild type and 2,327,100 from the *ago6-4* mutant, 2,846,684 (wt) and 1,261,189 (*ago6-4*) reads matched the genome, excluding 362,400 (wt) and 207,909 (*ago6-4*) that matched tRNA, rRNA, snRNA or snoRNAs.

### Methylation analysis

Methylation of the target enhancer and 5S rDNA repeats was analyzed, respectively, by bisulfite sequencing and methylation-sensitive restriction enzymes as described previously [13], [20], [24].

### GUS assays

GUS assays were carried out as described previously [35].

## References

[1] Matzke M, Kanno T, Daxinger L, Huettel B, Matzke AJM (2009). RNA-mediated chromatin-based silencing in plants.. Curr Opin Cell Biol.

[2] Law JA, Jacobsen SE (2010). Establishing, maintaining and modifying DNA methylation patterns in plants and animals.. Nat Rev Genet.

[3] He XJ, Chen T, Zhu JK (2011). Regulation and function of DNA methylation in plants and animals.. Cell Res.

[4] Wierzbicki A, Ream TS, Haag JR, Pikaard CS (2009). RNA polymerase V transcription guides ARGONAUTE4 to chromatin.. Nat Genet.

[5] Greenberg MVC, Ausin I, Chan SWL, Cokus SJ, Cuperus JT (2011). Identification of genes required for *de novo* methylation in *Arabidopsis*.. Epigenetics.

[6] Naumann U, Daxinger L, Kanno T, Eun C, Long Q (2011). Genetic evidence that DNA methyltransferase DRM2 has a direct catalytic role in RNA-directed DNA methylation in *Arabidopsis thaliana*.. Genetics.

[7] Cokus SJ, Feng S, Zhang X, Chen Z, Merriman B (2008). Shotgun bisulphite sequencing of the *Arabidopsis* genome reveals DNA methylation patterning.. Nature.

[8] Lister R, O'Malley RC, Tonti-Filippini J, Gregory BD, Berry CC (2008). Highly integrated single-base resolution maps of the epigenome in *Arabidopsis.*. Cell.

[9] Tsukahara S, Kobayashi A, Kawabe A, Mathieu O, Miura A (2009). Bursts of retrotransposition reproduced in *Arabidopsis*.. Nature.

[10] Saze H, Kakutani T (2011). Differentiation of epigenetic modifications between transposons and genes.. Curr Opin Plant Biol.

[11] Mirouze M, Reinders J, Bucher E, Nishimura T, Schneeberger K (2009). Selective epigenetic control of retrotransposition in *Arabidopsis*.. Nature.

[12] Ito H, Gaubert H, Bucher E, Mirouze M, Vaillant I (2011). An siRNA pathway prevents transgenerational retrotransposition in plants subjected to stress.. Nature.

[13] Daxinger L, Kanno T, Bucher E, van der Winden J, Naumann U (2009). A stepwise pathway for biogenesis of 24-nt secondary siRNAs and spreading of DNA methylation.. EMBO J.

[14] Ender C, Meister, G (2010). Argonaute proteins at a glance.. J Cell Sci.

[15] Mallory A, Vaucheret H (2010). Form, function, and regulation of ARGONAUTE proteins.. Plant Cell.

[16] Czech B, Hannon GJ (2011). Small RNA sorting: matchmaking for Argonautes.. Nat Rev Genet.

[17] Zilberman D, Cao X, Jacobsen SE (2003). ARGONAUTE4 control of locus-specific siRNA accumulation and DNA and histone methylation.. Science.

[18] He XJ, Hsu YF, Pontes O, Zhu J, Lu J (2009). NRPD4, a protein related to the RPB4 subunit of RNA polymerase II, is a component of RNA polymerases IV and V and is required for RNA-directed DNA methylation.. Genes Dev.

[19] Zheng X, Zhu JK, Kapoor A, Zhu JK (2007). Role of *Arabidopsis* AGO6 in siRNA accumulation, DNA methylation and transcriptional gene silencing.. EMBO J 26;.

[20] Kanno T, Bucher E, Daxinger L, Huettel B, Böhmdorfer G (2008). A structural-maintenance-of-chromosomes hinge domain-containing protein is required for RNA-directed DNA methylation.. Nat Genet.

[21] Vaucheret H (2008). Plant ARGONAUTES.. Trends Plant Sci.

[22] Takeda A, Iwasaki S, Watanabe T, Utsumi M, Watanabe Y (2008). The mechanism selecting the guide strand from small RNA duplexes among Argonaute proteins.. Plant Cell Physiol.

[23] Havecker E, Wallbridge LM, Hardcastle TJ, Bush MS, Kelly KA (2010). The *Arabidopsis* RNA-directed DNA methylation Argonautes functionally diverge based on their expression and interaction with target loci.. Plant Cell.

[24] Kanno T, Bucher E, Daxinger L, Huettel B, Kreil DP (2010). RNA-directed DNA methylation and plant development require an IWR1-type transcription factor.. EMBO Rep.

[25] Olmedo-Monfil V, Durán-Figueroa N, Arteaga-Vázquez M, Demesa-Arévalo E, Autran D (2010). Control of female gamete formation by a small RNA pathway in *Arabidopsis*.. Nature.

[26] Calaroco JP, Martienssen RA (2011). Genome reprogramming and small interfering RNA in the *Arabidopsis* germline.. Curr Opin Genet Devel.

[27] Mosher R, Schwach F, Studholme D, Baulcombe DC (2008). Pol IVb influences RNA-directed DNA methylation independently of its role in siRNA biogenesis.. Proc Natl Acad Sci USA.

[28] Qi Y, He X, Wang XJ, Kohany O, Jurka J (2006). Distinct catalytic and non-catalytic roles of ARGONAUTE4 in RNA-directed DNA methylation.. Nature.

[29] Steiner FA, Okihara KL, Hoogstrate SW, Sijen T, Ketting RF (2008). RDE-1 slicer activity is required only for passenger-strand cleavage during RNAi in *Caenorhabditis elegans*.. Nat Struct Mol Biol.

[30] Clough SJ, Bent AF (1998). Floral dip: a simplified method for *Agrobacterium*-mediated transformation of *Arabidopsis thaliana*.. Plant J.

[31] Taylor B, Powell A (1982). Isolation of plant DNA and RNA.. Focus.

[32] Li H, Durbin R (2009). Fast and accurate short read alignment with Burrows-Wheeler transform.. Bioinformatics.

[33] Li H, Handsaker B, Wysoker A, Fennell T, Ruan J (2009). The sequence alignment/map format and SAMtools.. Bioinformatics.

[34] Lu C, Meyers BC, Green PJ (2007). Construction of small RNA cDNA libraries for deep sequencing.. Methods.

[35] Stangeland B, Salehian Z (2002). An improved clearing method for GUS Assay in *Arabidopsis* endosperm and seeds.. Plant Mol Biol Rep.

